# Role of *Jnk1* in development of neural precursors revealed by iPSC modeling

**DOI:** 10.18632/oncotarget.11377

**Published:** 2016-08-18

**Authors:** Qian Zhang, Jian Mao, Xiaoxi Zhang, Haifeng Fu, Siyuan Xia, Zhinan Yin, Lin Liu

**Affiliations:** ^1^ Department of Cell Biology and Genetics, State Key Laboratory of Medicinal Chemical Biology, 2011 Collaborative Innovation Center for Biotherapy, College of Life Sciences, Nankai University, Tianjin, China

**Keywords:** Jnk1, induced pluripotent stem cells (iPSCs), modeling disease, neural differentiation, neural precursors

## Abstract

*Jnk1*-deficient mice manifest disrupted anterior commissure formation and loss of axonal and dendritic microtubule integrity. However, the mechanisms and the specific stages underlying the developmental defects remain to be elucidated. Here, we report the generation of *Jnk1*-deficient (*Jnk1* KO) iPSCs from *Jnk1* KO mouse tail-tip fibroblasts (TTFs) for modeling the neural disease development. The efficiency in the early induction of iPSCs was higher from *Jnk1* KO fibroblasts than that of wild-type (WT) fibroblasts. These *Jnk1* KO iPSCs exhibited pluripotent stem cell properties and had the ability of differentiation into general three embryonic germ layers *in vitro* and *in vivo*. However, *Jnk1* KO iPSCs showed reduced capacity in neural differentiation in the spontaneous differentiation by embryoid body (EB) formation. Notably, by directed lineage differentiation, *Jnk1* KO iPSCs specifically exhibited an impaired ability to differentiate into early stage neural precursors. Furthermore, the neuroepitheliums generated from *Jnk1* KO iPSCs appeared smaller, indicative of neural stem cell developmental defects, as demonstrated by teratoma tests *in vivo*. These data suggest that *Jnk1* deficiency inhibits the development of neural stem cells/precursors and provide insights to further understanding the complex pathogenic mechanisms of JNK1-related neural diseases.

## INTRODUCTION

The c-Jun NH_2_-terminal kinase (JNK) is a member of the mitogen-activated protein (MAP) kinase group of signaling proteins, which is encoded by two ubiquitously expressed genes (*Jnk1* and *Jnk2*) and by a tissue-specific gene (*Jnk3*), restricted to brain, heart and testis [1]. JNK plays a role in phosphorylating proteins and participates in a variety of biological processes, including migration, proliferation, differentiation and apoptosis, following responses to various stress stimuli [2–4]. Mice with deficiency of both *Jnk1* and *Jnk2* are embryonic lethal and exhibit defective neural tube closure caused by deregulated neural apoptosis [5, 6].

Although mice bearing homozygous disruption of any single *Jnk* gene are viable due to the compensative roles of JNKs, *Jnk1*-deficient mice display degeneration of anterior commissure and disorganized neuronal microtubules [7], as well as abnormal dendritic architecture [8], suggesting an indispensable role of JNK1 in neural development. In neurons, JNK1 is a major MAP2 (MT-associated protein 2) kinase, thus *Jnk1* deficiency results in hypophosphorylation and reduced ability of MAP2 to promote tubulin polymerization, associated with degeneration of microtubules [7]. Furthermore, JNK1 is mainly responsible for phosphorylation of the stathmin family microtubule-destabilizing protein SCG10 and suppresses its microtubule depolymerizing activity, contributing to microtubule homeostasis and axodendritic growth during brain development [9]. Maintenance of dendrite homeostasis is important for normal neuronal physiology, and dysregulation of dendritic structure is a hallmark of schizophrenia, autism and mental retardation syndromes, such as Rett syndrome and Down's syndrome, in which abnormality in length and branching of dendritic arbors is observed [10–14]. Therefore, understanding the mechanisms of dendrite formation and homeostasis, which is related to JNK1, may provide important clues to the etiology of such diseases.

Induced pluripotent stem cells (iPSCs), derived from somatic cells by reprogramming with defined exogenous transcription factors [15, 16] and/or small molecular compounds [17, 18], maintain features similar to those of embryonic stem cells (ESCs). iPSCs have the ability to self-renew and generate various cell types in the body, indicating true pluripotency [19–21]. Therefore, iPSCs may represent an alternative to ESCs as a cell resource applied in regenerative therapy and provide an applicable platform to mimic the pathogenic process of diseases [22–26]. Herein, we established *Jnk1*-deficient (*Jnk1* KO, *Jnk1*^−/−^) iPSCs from *Jnk1* KO mouse tail-tip fibroblasts (TTFs) in an attempt to model the neural disease development, and interestingly found impaired ability of *Jnk1* KO iPSCs in undergoing neural differentiation *in vitro* and *in vivo*, especially with defects in generation of neural precursors, compared with wild-type (WT) iPSCs.

## RESULTS

### *Jnk1* deficiency enhances early induction of iPSCs

**Figure 1:**
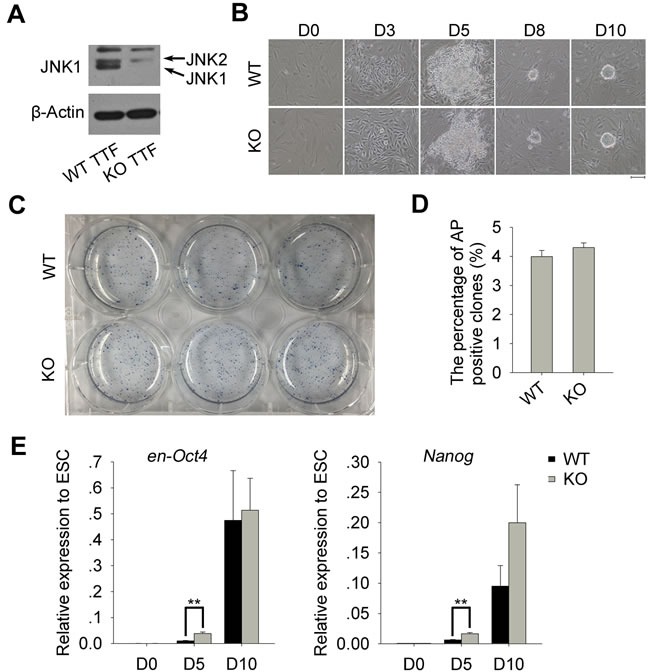
Derivation of iPSCs from *Jnk1* KO and WT TTFs **A.** Confirmation of JNK1 deficiency in *Jnk1* KO TTFs by western blot. **B.** Morphological changes of TTFs during the induction of iPSCs and their primary ESC-like clones. Scale bar = 100 μm. **C.** The alkaline phosphatase (AP) staining of primary iPS clones derived from TTFs on day 13. **D.** Induction efficiency of primary iPS clones estimated by AP activity assay, based on number of cells (0.34×10^4^) per well plated on day 5. No statistical difference (*P* > 0.05) *n* = 3. **E.** Relative expression levels of reactivated endogenous pluripotency-associated genes, *en-Oct4* and *Nanog*, during reprogramming by qPCR analysis. N33 ESCs served as positive control. **P < 0.01, *n* = 4.

First, we isolated and generated TTFs from *Jnk1* KO and WT C57BL/6 mice, and confirmed their genotypes by western blot (Figure 1A). Then, we generated iPSCs from *Jnk1* KO and WT TTFs by retroviral transduction with four Yamanaka factors, Oct4, Sox2, Klf4 and c-Myc (OSKM) [15, 27]. Cells began to aggregate around day 3 post-infection, and colony aggregates formed on day 5, followed by plating on inactivated MEFs as feeders. ESC-like colonies, with a round shape and distinct edge, formed on day 10, regardless of *Jnk1* deficiency (Figure 1B). The primary iPS clones showed alkaline phosphatase (AP)-positive staining (Figure 1C), and the percentage of AP-positive clones exhibited no significant difference between *Jnk1* KO and WT cells (Figure 1D). We also examined endogenous expression of pluripotency-associated genes, *en-Oct4* and *Nanog*, during reprogramming by quantitative real-time PCR (qPCR) analysis (Figure 1E). Expression levels of *en-Oct4* and *Nanog* did not differ between WT cells on day 5 of induction and TTFs, whereas *Jnk1* KO cells expressed higher levels of *endo-Oct4* and *Nanog* than did WT cells, suggesting that *Jnk1* deficiency promotes endogenous pluripotent genes reactivating early during somatic cell reprogramming.

### *Jnk1* KO iPSCs exhibit pluripotent stem cell properties

**Figure 2:**
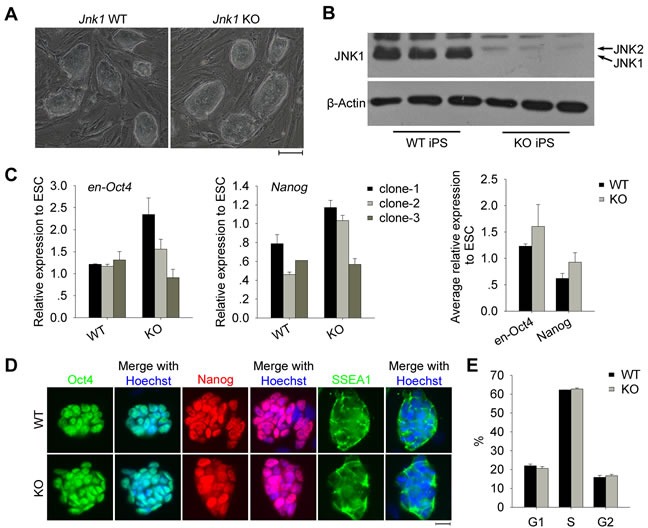
Characterization of *Jnk1* KO iPSCs **A.** Morphology of *Jnk1* KO iPSCs and WT iPSCs. Scale bar = 100 μm. **B.** Confirmation of JNK1 deficiency in *Jnk1* KO iPSCs by western blot. **C.** Relative expression levels of endogenous pluripotency-associated genes (*en-Oct4* and *Nanog*) in iPSCs at passage 7 by qPCR analysis. N33 ESCs served as positive control. **D.** Immunofluorescence staining of pluripotency markers (Oct4, Nanog and SSEA1) in *Jnk1* KO and WT iPSCs. Scale bar = 20 μm. **E.** Cell-cycle analysis by flow cytometry showed no significant difference between *Jnk1* KO and WT iPSCs. *n* = 3.

We obtained stably passaged iPSC lines generated from *Jnk1* KO and WT TTFs. These stable iPSCs maintained characteristics of ESCs in morphology, displaying large nuclei and nucleoli under higher magnification with clear compact clonal boundaries, distinct from feeder fibroblasts (Figure 2A). *Jnk1* deficiency was confirmed in *Jnk1* KO iPSCs by western blot (Figure 2B). *Jnk1* KO iPSCs and WT iPSCs expressed similar high levels of endogenous *Oct4* (*en-Oct4*) and *Nanog* shown by qPCR analysis (Figure 2C). The pluripotency of *Jnk1* KO and WT iPSC clones also was confirmed by immunofluorescence assay, showing Oct4 and Nanog in the nuclei, and SSEA1 on the cell surface (Figure 2D). Furthermore, *Jnk1* KO iPSCs displayed proliferation progression similar to that of WT iPSCs by cell-cycle analysis (Figure 2E), so did other *Jnk1* KO and WT iPSC lines (Supplementary Figure 1). These data suggested that *Jnk1* does not affect expression of pluripotency genes and proliferation of iPSCs.

### *Jnk1* KO iPSCs can differentiate into three embryonic germ layers *in vitro* and *in vivo*

**Figure 3:**
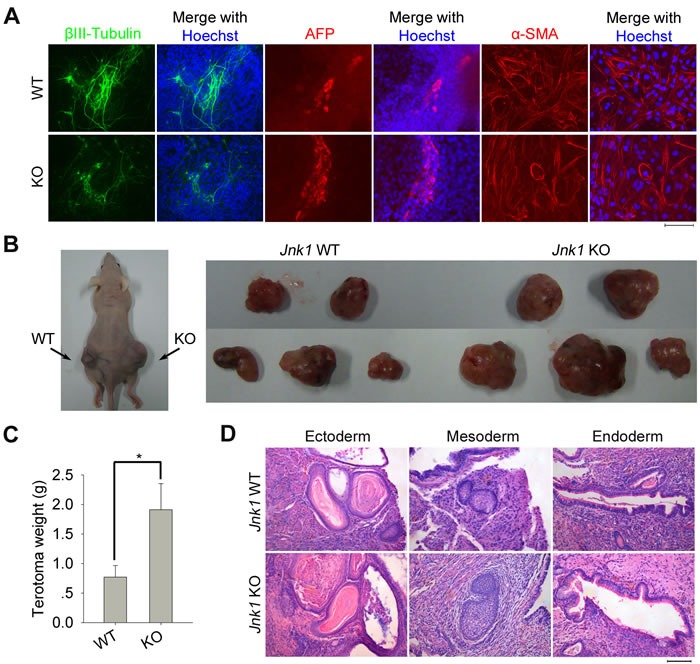
Differentiation of *Jnk1* KO iPSCs *in vitro* and *in vivo* **A.** Differentiation *in vitro* of *Jnk1* KO and WT iPSCs by embryoid body (EB) formation. The differentiated derivatives consist of cells representing three embryonic germ layers as indicated by immunofluorescence staining of markers for ectoderm (βIII-Tubulin), endoderm (AFP) and mesoderm (α-SMA). Scale bar = 100 μm. **B.** Differentiation *in vivo* of *Jnk1* KO and WT iPSCs by teratoma formation test following injection into nude mice. Black arrows indicate teratomas on the back of nude mice. **C.** The teratomas formed from *Jnk1* KO iPSCs showed heavier than those of WT iPSCs by statistics. *P < 0.05, *n* = 5. **D.** Hematoxylin and eosin staining of teratoma tissues derived from *Jnk1* KO and WT iPSCs. All teratomas consist of representative derivatives of three germ layers, including epidermis (ectoderm), cartilage (mesoderm) and gland epithelium (endoderm).

To examine the developmental potential of *Jnk1* KO iPSCs, we performed *in vitro* differentiation by standard embryoid body (EB) formation assay. Differentiation of *Jnk1* KO and WT iPSCs via EB formation both yielded cells representing three embryonic germ layers as indicated by tissue-specific immunofluorescence staining of βIII-Tubulin (neurons, ectoderm), AFP (liver, endoderm) and α-SMA (cardiac muscle, mesoderm) (Figure 3A). The *in vivo* differentiation was tested by teratoma formation following transplantation into nude mice, and *Jnk1* KO and WT iPSCs all formed teratomas (Figure 3B). The size and weight of teratomas derived from *Jnk1* KO iPSCs were larger and heavier than those of WT iPSCs (Figure 3C), suggesting that *Jnk1* KO iPSCs may have higher cell growth and proliferation *in vivo* differentiation. The teratomas of *Jnk1* KO and WT iPSCs all displayed representative derivatives of three germ layers, including epidermis (ectoderm), cartilage (mesoderm) and gland epithelium (endoderm; Figure 3D). These *in vitro* and *in vivo* characterizations demonstrated that *Jnk1* KO iPSCs closely resemble WT iPSCs in terms of pluripotency and differentiation potential.

### *Jnk1* KO iPSCs exhibit defects in neural differentiation *in vitro* and particularly in generation of neural precursors

**Figure 4:**
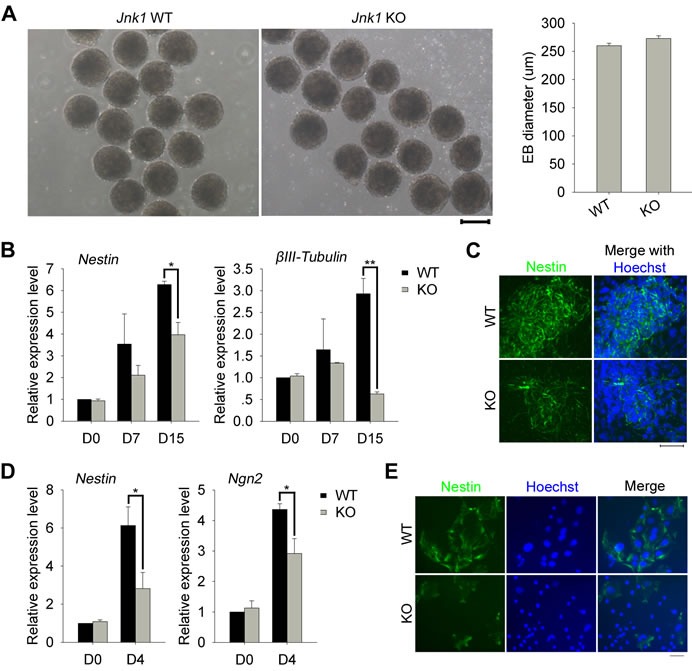
*Jnk1* KO iPSCs exhibited defects in neural differentiation *in vitro* **A.** Embryoid body (EB) formation of *Jnk1* KO and WT iPSCs on day 4, and average size of EBs was calculated by ImageJ software. Scale bar = 200 μm. *n* = 40. **B.** Neural marker genes, *Nestin* and *βIII-Tubulin*, showed reduced expression in differentiation from *Jnk1* KO iPSCs by qPCR analysis. *n* = 3. **C.** Immunofluorescence analysis of Nestin on day 15, using other *Jnk1* KO iPSC and WT iPSC lines, confirmed reduced Nestin rosettes in the differentiation of *Jnk1* KO iPSCs. Scale bar = 50 μm. **D.** qPCR analysis showed decreased expression levels of *Nestin* and *Ngn2* on day 4 of neural induction from *Jnk1* KO iPSCs compared with those of WT iPSCs. *n* = 4. **E.** Immunofluorescence analysis of Nestin on day 8 indicated impairment of forming neural precursor colonies from *Jnk1* KO iPSCs. Scale bar = 50 μm. *P < 0.05, **P < 0.01, compared with WT controls.

Using standard *in vitro* differentiation test by EB formation, we observed no noticeable effects of *Jnk1* deficiency on size of EBs on day 4 (Figure 4A). We also measured expression of three embryonic germ layer marker genes at various time points during EB differentiation by qPCR analysis. *Jnk1* deficiency did not impact expression levels of *Sox17* and *Gata4* (endoderm), nor that of *T* (*Brachyury*) and *Flk1* (mesoderm; Supplementary Figure 2A and 2B). Furthermore, expression levels of *Fgf5* (ectoderm) also showed no significant difference between *Jnk1* KO EBs and WT EBs (Supplementary Figure 2C). However, *Jnk1* deficiency resulted in significantly decreased expression of neural marker genes, *Nestin* and *βIII-Tubulin* (Figure 4B), consistent with immunostaining data of βIII-Tubulin shown above (Figure 3A). We also performed immunofluorescence analysis of Nestin in other *Jnk1* KO iPSC and WT iPSC lines. Consistently, compared with WT iPSCs, which efficiently formed neural rosettes of Nestin on day 15, *Jnk1* KO iPSCs exhibited reduced Nestin rosettes in EB differentiation (Figure 4C).

To further confirm the role of *Jnk1* in neural differentiation, we generated a relatively homogenous population of neural precursors from both *Jnk1* KO iPSCs and WT iPSCs based on a method of adherent monoculture published previously [28]. Expression of *Nestin*, marker of neural stem cells/precursors [29, 30], and *Ngn2*, which promotes neuronal differentiation of neural progenitor cells [31], was significantly reduced on day 4 during neural induction of *Jnk1* KO iPSCs compared with that of WT iPSCs, by qPCR analysis (Figure 4D). In contrast to compact colonies of WT neural precursors expressing Nestin, *Jnk1* KO iPSCs failed to efficiently form neural precursor colonies, with only sporadical expression of Nestin revealed by immunofluorescence microscopy (Figure 4E). These data indicate that *Jnk1* deficiency reduces differentiation into neural precursors *in vitro*. We also measured expression of genes, *JunD* and *Fra2*, which are involved in JNK signalling pathway [32–34], by qPCR analysis (Supplementary Figure 3). The decreased expression of *JunD* and *Fra2* during neural induction of *Jnk1* KO iPSCs, suggested that JNK1 pathway was involved in the differentiation of iPSCs into early stage neural precursors.

### *Jnk1* KO iPSCs show impaired development of neural precursors *in vivo*

**Figure 5:**
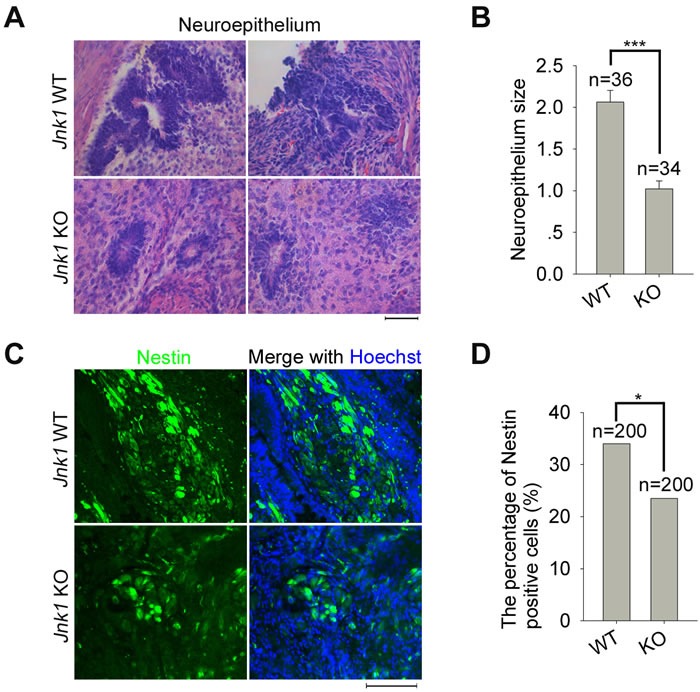
*Jnk1* KO iPSCs showed impaired ability in development of neural precursors *in vivo* **A.** Morphology of neuroepitheliums in teratomas generated from *Jnk1* KO and WT iPSCs. Scale bar = 50 μm. **B.** The average size of neuroepitheliums calculated by ImageJ software showed decreased significantly in teratomas of *Jnk1* KO iPSCs compared to those of WT iPSCs. n, number of neuroepitheliums counted. **C.** Immunofluorescence analysis of Nestin in teratomas of *Jnk1* KO iPSCs and WT iPSCs. Scale bar = 100 μm. **D.** The percentage of Nestin positive cells in Nestin expressed areas reduced in teratomas of *Jnk1* KO iPSCs. n, number of cells counted, based on 10 images of each group. χ^2^ test. *P < 0.05, ***P < 0.001, compared with WT controls.

To validate the findings obtained by neural differentiation *in vitro*, we also tested neural differentiation *in vivo* by teratoma formation. The rosette neuroepitheliums in teratomas derived from *Jnk1* KO iPSCs, exhibited a significantly decreased average size compared to that of WT iPSCs (Figure 5A and 5B). WT iPSCs efficiently generated regular large neural rosettes shown by immunofluorescence of Nestin, whereas *Jnk1* KO iPSCs developed to smaller, irregular or uncompact neural rosettes (Figure 5C). Moreover, the percentage of Nestin positive cells in Nestin expressed areas of teratomas, derived from *Jnk1* KO iPSCs, was reduced compared to that of WT iPSCs (Figure 5D), further supporting the notion that *Jnk1* deficiency reduces formation of neural precursors in neuroepitheliums.

## DISCUSSION

We have established *Jnk1* KO iPSCs from TTFs for modeling neural differentiation defects. The implication of iPSC induction from adult cells (here TTFs) is that these cells are readily accessible and thus can better fit to the purpose of clinical application, unlike embryonic fibroblasts (MEFs) which have to be isolated from fetus. A previous report showed that *Jnk1* KO MEFs exhibit greater potency in increasing AP-positive iPSC colonies compared to that of WT MEFs [35]. The percentage of AP-positive clones induced from TTFs remained no significant difference between *Jnk1* KO cells and WT cells. However we find that endogenous *Oct4* and *Nanog* are reactivated earlier and expressed higher in the induction of *Jnk1* KO iPSCs compared with that of WT iPSCs. These data together suggest a negative role of *Jnk1* in iPSC reprogramming. A most recent report shows that RNAi mediated downregulation of *JNK1* results in complete abrogation of hiPSC colony formation and emergence of partially reprogrammed colonies which are lost during the maturation stage of reprogramming [36]. In addition to the species differences, knockout in our cells completely deletes *Jnk1*, whereas shRNA-mediated knock-down strategy only partly reduces *JNK1* expression and also may bring off-target effects.

Interestingly, we find that *Jnk1* KO iPSCs suffer from defects in neural differentiation, especially in generation of neural precursors, although they possess normal pluripotent stem cell properties. Earlier, JNK also was found not required for self-renewal or proliferation of mouse ESCs, but required for lineage-specific differentiation [2, 37]. The cell number of embryoid bodies (EBs) derived from *Jnk1* KO ESCs was similar to that of WT ESCs on day 3 of EB formation, but increased on day 8, suggesting a stage-specific role of *Jnk1* in proliferation and that *Jnk1* deficiency may enhance proliferation at late stage of differentiation [37]. We also find that the size of EBs does not differ between *Jnk1* KO and WT cells on day 4 of differentiation, yet teratomas derived from *Jnk1* KO iPSCs are larger than those of WT iPSCs by 4 weeks.

JNK binds to a large set of active promoters, which are enriched with binding motifs for the transcription factor NF-Y, and phosphorylates their histone H3 Ser10 (H3S10) during the conversion of stem cells into neurons, enhancing expression of target genes and neural differentiation [38]. Previous report indicated inhibited neurogenesis in *Jnk1*-deficient ESCs, with reduced expression of neuron marker, neurofilament light chain (NFLC) [39]. We find neural precursor/stem cell differentiation defects of *Jnk1* KO iPSCs as shown by reduced expression levels of Nestin, marker of neural precursors [29, 30], and impaired rosette formation, indicating that *Jnk1* deficiency inhibits the development of neural precursors. Furthermore, from the UCSC Genome Browser on Mouse, the promoter of *Nestin* possesses the JNK-bound NF-Y-like motif, suggesting that *Jnk1* can increase the transcription of *Nestin* by activating its promoter activity.

In conclusion, by iPSC technology to model diseases using adult mouse cells with *Jnk1* deficiency, we unexpectedly find a novel positive role of *Jnk1* in the development of neural precursors/stem cells. While *Jnk1* is dispensable for pluripotency maintaining and self-renewal of iPSCs, *Jnk1* KO iPSCs exhibited defects in neural differentiation, especially generation of neural precursors, both *in vitro* and *in vivo*, providing a new model to further study the pathogenesis of JNK1-related neural diseases. However, the neural differentiation of *Jnk1* KO iPSCs in our study remains at primary stage, which needs further lineage-directed differentiation into mature neurons to model the diseases.

## MATERIALS AND METHODS

### iPSC induction

The care and use of mice for this research were based on the guidelines and protocols for the animal research approved by the Institutional Animal Care and Use Committee (IACUC) of Nankai University.

Isolation and generation of *Jnk1* KO and WT TTFs from C57BL/6 adult mouse tail-tips were performed as described previously [40]. iPSCs were induced by transduction with four Yamanaka factors using a standard protocol [41], with slight modification. Reprogrammed pluripotent cells can be isolated from genetically unmodified somatic donor cells solely based on morphological criteria [42]. Briefly, Plat-E cells were seeded at 5×10^6^ cells per 100-mm dish. On the next day, pMXs-based retroviral vectors (pMXs-*Oct4*, *Sox2*, *Klf4* and *c-Myc*) were introduced into Plat-E cells using lipo-2000 transfection reagent according to the manufacturer's recommendations. Viruses were collected through 0.45 μm filter membrane at 48 h and 72 h after transfection. For iPSC induction, TTFs were seeded at 4×10^4^ cells per well of six-well dish 24 h before infection, and were infected by fresh collected viruses twice at 24 h interval. From day 0 to day 2 post-infection, cells were cultured in ESC medium containing knock-out DMEM (Invitrogen) supplemented with 20% FBS (Hyclone), 1000 U/ml mouse leukemia inhibitory factor (LIF; ESG1107, Millipore), 0.1 mM non-essential amino acids, 0.1 mM b-mercaptoethanol, 1 mM L-glutamine, penicillin (100 U/ml) and streptomycin (100 mg/ml). And then 20% FBS was replaced with 20% knock-out serum replacement (Invitrogen) in medium to better reprogram cells. Five days after infection, the cells were passaged on MEF feeders and the medium was changed daily. Thirteen days after infection, ESC-like colonies were picked and passaged using standard protocols. For alkaline phosphatase (AP) assay, 3400 cells were plated in a six-well plate, and the positive colonies assessed using the Vector Blue Kit (SK-5300, Vector Laboratories).

### iPSC and ESC culture

N33 ESC line was derived from C57BL/6 mice [40] and served as positive control. The ESC and iPSC culture medium consisted of knock-out DMEM (Invitrogen) with 20% FBS (Hyclone), 1000 U/ml mouse LIF (ESG1107, Millipore), 0.1 mM non-essential amino acids, 0.1 mM β-mercaptoethanol, 1 mM L-glutamine, penicillin (100 U/ml) and streptomycin (100 mg/ml). For culture of iPSCs and ESCs, the medium was changed daily, and cells were routinely passaged every two days.

### Directed neural lineage differentiation

The directed neural lineage differentiation from iPSCs based on adherent monoculture was performed as described previously [28] with slight modification. Undifferentiated iPSCs were trypsin digested and plated onto 0.1% gelatin-coated six-well dish at a density of 1-2×10^4^/cm^2^ in normal iPSC culture medium. On the next day (day 0), when cells adhered to the dish, the medium was changed to N2B27 medium, which is a 1:1 mixture of DMEM/F12 (11330032, Life technologies) supplemented with N2 (17502048, Life technologies) and Neurobasal medium (12348017, Life technologies) supplemented with B27 (12587010, Life technologies), and added with 50 μg/ml bovine serum albumin (BSA; Sigma). Medium was renewed every two days.

### Embryoid body formation test

iPSCs were removed off feeders twice based on their differences in the adherence to the bottom of dish. The cells were diluted to 4×10^4^ per milliliter and every 30 ml was pipetted to form a hanging drop on the cover of 100-mm dish. Embryoid bodies (EBs) formed on day 4, and then were transferred to six-well plates for adherent culture. EBs were fixed for immunofluorescence staining using markers of three embryonic germ layers on day 15.

### Teratoma test

1×10^6^ iPSCs were injected subcutaneously into about 6-week-old immunodeficient nude mice. About 4 weeks after injection, the mice were humanely sacrificed, and the teratomas were excised, fixed in 4% paraformaldehyde, dehydrated in gradient ethanol, embedded in paraffin, and sectioned for histological examination by haematoxylin and eosin staining.

### Gene expression analysis by quantitative real-time PCR

Total RNA was purified using a RNA mini kit (Qiagen), treated with DNase I (Qiagen), and the cDNA was generated from 2 g RNA using Oligo(dT) 18 primer (Takara) and M-MLV Reverse Transcriptase (Invitrogen). Real-time quantitative PCR reactions were set up in duplicate with the FS Universal SYBR Green Master (Roche) and carried out on an iCycler MyiQ2 Detection System (BIO-RAD). All reactions were carried out by amplifying target genes and internal control gene (GAPDH) in the same plate. The amplification was performed for primary denaturation at 95°C for 10 min, then 40 cycles of denaturation at 95°C for 15 s, annealing and elongation at 58°C for 1 min, and the last cycle under 55-95°C for dissociation curve. Relative quantitative evaluation of target gene was determined by comparing the threshold cycles. Primers were confirmed their specificity with dissociation curves. Most primers were designed using the IDT DNA website and primers used are listed in Supplementary Table S1.

### Western blot

Cells were washed twice in PBS, collected, and lysed in cell lysis buffer on ice for 30 min and then sonicated for 1 min at 60 of amplitude with 2 s intervals. After centrifugation at 10,000 g, 4°C for 10 min, supernatant was transferred into new tubes. The concentration of the protein sample was measured by bicinchoninic acid, and then protein samples were boiled in SDS Sample Buffer at 99°C for 10 min. 20 μg total proteins of each cell extracts were resolved by 10% Bis-Tris SDS-PAGE and transferred to polyvinylidene difluoride membranes (PVDF; Millipore). Nonspecific binding was blocked by incubation in 5% skim milk in TBST at room temperature for 2 h. Blots were then probed with primary antibodies, JNK1 (SGA0288, Sungene) and β-Actin (P30002, Abmart) by overnight incubation at 4°C in 5% skim milk in TBST. Immunoreactive bands were then probed for 2 h at room temperature with the appropriate horseradish peroxidase (HRP)-conjugated secondary antibodies, anti-Rabbit IgG-HRP (NA934V, GE Healthcare). Protein bands were detected by Chemiluminescent HRP substrate (WBKLS0500, Millipore).

### Immunofluorescence microscopy

Cells or tissue sections were washed twice in PBS, fixed in freshly prepared 3.7% paraformaldehyde for 30 min at 4°C, washed once in PBS and permeabilized in 0.1% Triton X-100 in blocking solution (3% goat serum plus 0.1% BSA in PBS) for 30 min at room temperature, then washed once in PBS, and left in blocking solution for 2 h. Cells were incubated overnight at 4°C with primary antibodies against Oct4 (sc5279, Santa Cruz), Nanog (ab80892, Abcam), SSEA-1 (MAB4301, Millipore), βIII-Tubulin (CBL412, Chemicon), alpha 1-fetoprotein (AFP; DAK-N1501, Dako), alpha smooth muscle actin (α-SMA; ab5694-100, Abcam) and Nestin (MAB353, Millipore). Then cells were washed three times (each for 15 min) with blocking solution, and incubated for 2 h with secondary antibodies at room temperature. Goat Anti-Mouse IgG (H+L) FITC (115-095-003, Jackson) and Goat Anti-Rabbit IgG (H+L) Alexa Fluor^®^ 594 (111-585-003, Jackson), diluted 1:200 with blocking solution, were used. Samples were washed, and counterstained with 0.5 mg/ml Hoechst 33342 (H1398, MP) in Vectashield mounting medium. Fluorescence was detected and imaged using a Zeiss Axio-Imager Z1 fluorescence microscope.

### Cell-cycle analysis

Single-cell suspensions were prepared by trypsinization and washed once in cold PBS. iPSCs were fixed in ice-cold 70% (vol/vol) ethanol and incubated overnight at 4°C. Following RNase A treatment, total DNA was stained with propidium iodide (Beyotime) at 37°C for 30 min. Cells were analyzed with a Flow Cytometer (BD FACS Calibur) and the data were processed using FlowJo.

### Statistical analysis

All results were analyzed by student's *t*-test or χ^2^ test (specially mentioned) and the resulting *P*-values were shown. Significant differences were defined as *P < 0.05, **P < 0.01, or ***P < 0.001. The results were shown as mean ± SEM.

## SUPPLEMENTARY MATERIALS FIGURES AND TABLE


