# Supplementary material for: Role of Jnk1 in development of neural precursors revealed by iPSC modeling
Source: Oncotarget. 2016 Aug 18;7(38):60919–28. doi: 10.18632/oncotarget.11377 (PMC5308626; doi:10.18632/oncotarget.11377)
Supplement: Supplementary file 1 [file oncotarget-07-60919-s001.pdf]

Role of *Jnk1* in development of neural precursors revealed by iPSC modeling

Supplementary Materials

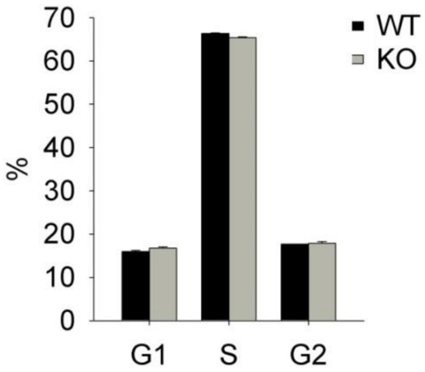

Supplementary Figure S1: Cell-cycle analysis by flow cytometry. Cell-cycle analysis of other *Jnk1* KO iPSC and WT iPSC lines showed no significant difference in the proliferation.

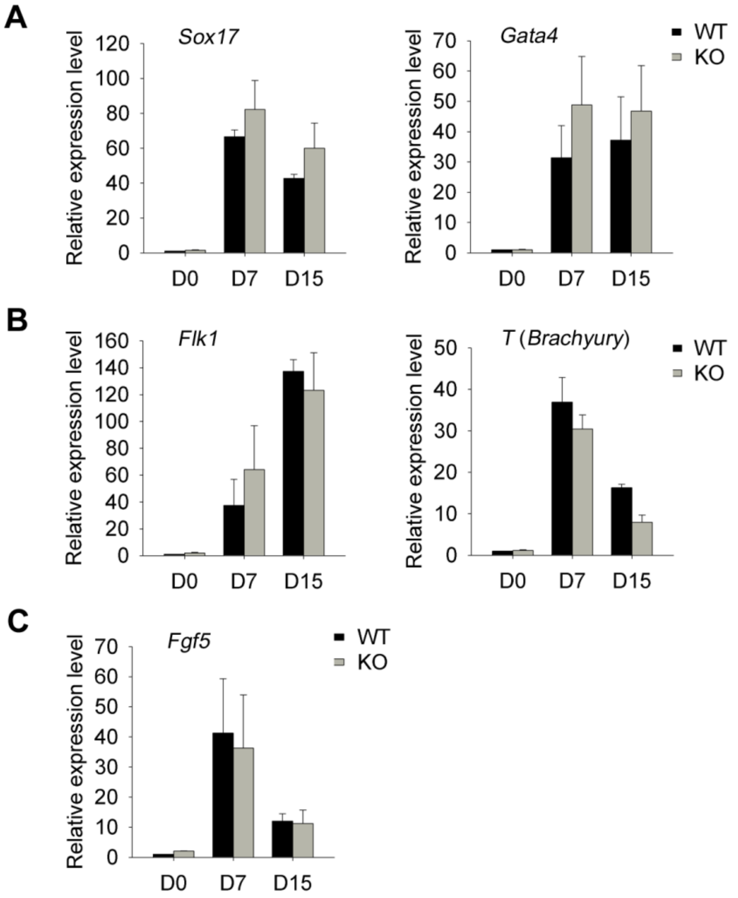

Supplementary Figure S2: Expression levels of three embryonic germ layer marker genes during EB formation by qPCR analysis. (**A**) Endoderm, *Sox17* and *Gata4*. (**B**) Mesoderm, *Flk1* and *T* (*Brachyury*). (**C**) Ectoderm, *Fgf5*. n = 3.

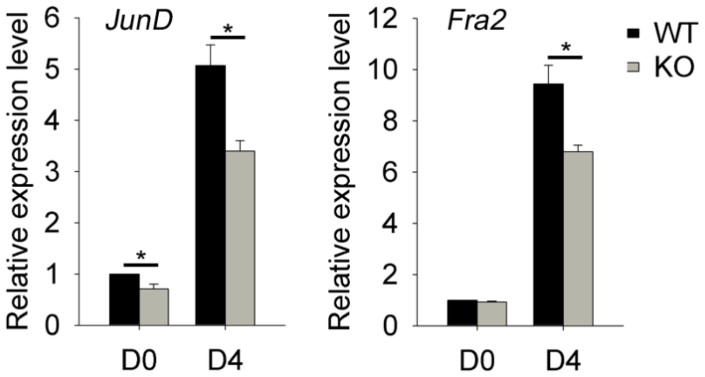

Supplementary Figure S3: Expression levels of *JunD* and *Fra2* during neural induction by qPCR analysis. Expression of *JunD* and *Fra2*, which are involved in JNK signalling pathway, decreased during neural induction of *Jnk1* KO iPSCs compared with that of WT iPSCs. n = 3.

Supplementary Table S1:

Primers used for quantitative real-time PCR analysis

Gene name	Primer
GAPDH	TCAACAGCAACTCCCACTCTTCCA<br>ACCACCCTGTTGCTGTAGCCGTAT
en-Oct4	TCTTTCCACCAGGCCCCCGGCTC<br>TGCGGGCGGACATGGGGAGATCC
Nanog	TTGCTTACAAGGGTCTGCTACT<br>ACTGGTAGAAGAATCAGGGCT
Sox17	CTCGGGGATGTAAAGGTGAA<br>GCTTCTCTGCCAAGGTCAAC
Gata4	GCTATGCATCTCCTGTCACTCAGA<br>CCAAGTCCGAGCAGGAATTTGAAG
T (Brachyury)	CTGGGAGCTCAGTTCTTTCG<br>CCCCTTCATACATCGGAGAA
Fgf5	GAAACTCGGATACAGCATCCCTCT<br>GGATCGCTACAGAGAATCCCACTT
Nestin	CCCTGAAGTCGAGGAGCTG<br>CTGCTGCACCTCTAAGCGA
βIII-Tubulin	GGCAACTATGTAGGGGACTCAG<br>CCTGGGCACATACTTGTGAG
Ngn2	CATGCACAACCTAAACGCC<br>CAGATGTAATTGTGGGCGAAG
JunD	CACAGATACACAACCACACA<br>ATCCTCTAGACTCCGCTATTC
Fra2	TCGCCGGGAGCTGACA<br>GCAGCTCAGCAATCTCTTTCTG
